# Diversity competence training for health professionals in Europe: a modified delphi study investigating relevant content for short or online courses

**DOI:** 10.1186/s12909-023-04563-z

**Published:** 2023-08-21

**Authors:** Janne Sorensen, Camilla Michaëlis, Julie Marie Møller Olsen, Allan Krasnik, Kayvan Bozorgmehr, Sandra Ziegler

**Affiliations:** 1 Danish Research Centre for Migration, Ethnicity and Health, Department of Public Health, University of Copenhagen, Oester Farimagsgade 5A, Copenhagen, DK-1353 Denmark; 2 Section for Health Equity Studies & Migration, Department of General Practice and Health Services Research, Department of Population Medicine and Health Services Research, School of Public Health, Heidelberg University Hospital, University of Bielefeld, Universitätsstraße 25, Bielefeld, 33615 Germany; 3 Section for Health Equity Studies & Migration, Department of General Practice and Health Services Research, Heidelberg University Hospital, Im Neuenheimer Feld 130.3, Heidelberg, 69120 Germany

**Keywords:** Diversity competence, Medical education, Short courses, Online courses, Delphi study, Training, Health professionals, Migrant health, Minority health, Continuing education

## Abstract

**Background:**

Diversity is a reality in our societies, requiring health professionals to adapt to the unique needs of all patients, including migrants and ethnic minorities. In order to enable health professionals to meet related challenges and reduce health disparities, long and demanding training courses have been developed. But due to busy schedules of professionals and often scarce resources, a need for shorter training courses exists. This study aims to investigate which topics and methods should be prioritised in designing basic diversity training courses that provide health professionals the opportunity to foster this competence.

**Methods:**

The study provided an expert panel of 31 academic and clinical migrant health experts with the content and methods of an existing diversity training course. The panel was asked to prioritise training topics and teaching methods in a two-stage process, using an adapted Delphi method. In the first stage, experts rated 96 predefined items, commented on those items, provided answers to eight open-ended questions and suggested additional content for a short course. In the second stage, they commented on the ratings from Round 1, and rated new suggested content. Consensus for training topics was set to 80% and for teaching methods 70%.

**Results:**

The entire panel deemed ‘health effects of migration (pre-, during- and post-migration risk factors)’ to be important or very important to include in a short/online, basic diversity training (100% consensus). Other high-scoring items and therefore topics to be included in trainings were ‘social determinants of health’ (97%) and ‘discrimination within the healthcare sector’ (also 97%). A general trend was to focus on reflective practice since almost all items regarding reflection reached consensus. ‘Reflection on own stereotypes and prejudices’ (97%) was the highest-rated reflection item. ‘Opportunities and best practices in working with interpreters’ was the highest-scoring skills item, both on consensus (96%) and mean value (5.77).

**Conclusions:**

Experts’ prioritizations of teaching content and methods for diversity training can help the design of short (online) trainings for health professionals and reduce unnecessary course content, thereby fostering professional development and enabling diversity competence trainings to be implemented also when time and/or financial resources are limited.

## Background

Diversity in our societies is a reality [1] requiring health systems to adapt to the specific needs of diverse patient groups. Evidence demonstrates health and healthcare access inequalities between minorities and the majority population [2–9]. For migrants and ethnic minorities (MEM) this has been further highlighted by the Covid-19 pandemic [10, 11]. Studies have documented suboptimal encounters between health professionals and MEM, which can be characterised – among other things – by insecurities on both sides, as well as different expectations regarding the health service encounter [12–14]. It is therefore important to understand which competences of health professionals are necessary to enhance so as to improve encounters with patients of, for instance, different socio-economic background, age, gender, sexual orientation, ethnicity or religion, in order to reduce health disparities and provide health services that meet the unique needs of all patients.

We use the term ‘diversity’ in this study to embrace the diversity *within* diversity – sometimes called ‘superdiversity’ [1], pointing to the multicollectivity [15, 16] of individual migrants and members of minorities and suggesting an intersectionality-oriented approach. Improving the intercultural competence of health professionals has been acknowledged as an important strategy to reduce health disparities among different MEM [17, 18], but accounting for the superdiversity of this population implies that opportunities to participate or dangers of being discriminated against can be determined by several, sometimes overlapping, ascriptions [19] that exceed mere ascriptions of culture or ethnicity.

In line with the definition that our experts specified in a related part of this study, we define ‘diversity competence for health professionals’ as a respectful, aware and self-reflective attitude, where professionals are conscious of social determinants of health, can communicate understandably and listen empathetically. They strive towards an individualised, equitable, ethical and human rights-based practice [20]. Diversity competence can contribute to improving the interaction, communication and understanding between health professionals and patients with individual diverse identities [21, 22].

The WHO and the European Union encourage capacity building in the form of diversity competence training in healthcare [23, 24]. Despite existing educational programmes, studies suggest that diversity competence is not yet adequately included in medical education [25, 26] and many health professionals experience barriers to engaging in continuing medical education (CME) due to limited time and priorities of funding, among other factors [27]. Short, basic trainings – possibly in online formats – could be a potential solution, as they would be independent of location and time and therefore provide flexibility for learners [28]. They could also be provided at lower cost than long, in-person training courses. Shorter courses, with an online option, could offer basic, easily accessible trainings, attracting more participants and inviting health professionals who would otherwise not attend such trainings.

Diversity education is a complex task, therefore longer, more extensive training programmes also need to be available. Designing shorter courses requires the prioritisation of a vast number of possible teaching objectives. Yet to ensure high-quality, accessible and equitable care for today’s diverse patients, and to close gaps in health professionals’ education [29], online CME on diversity competence would seem to suit, since this would accommodate professionals’ busy schedules, allowing flexibility at low cost. Thus, the objective of this study is to investigate which topics and methods should be prioritised in short, possibly online, courses on diversity competence in healthcare delivery with particular focus on MEM. The study was part of the EIT Health-financed project *Improving Diversity Sensitivity in Healthcare – Training for Health Professionals* (IMPRODISE) [30], which aimed to ensure high quality, accessible and equitable care for today´s diverse patient populations and close the respective gaps in professionals’ education [29].

## Methods

To investigate which topics and methods that should be prioritised in short and possibly online courses on diversity competence in healthcare delivery with particular focus on MEM, the Delphi method were chosen. The Delphi method is used to achieve consensus through a multi-staged survey process among a group of experts on a certain issue where no agreement previously existed [31]. Most Delphi studies consist of two or more rounds of ratings and discussions [31]. In this study it was decided to adapt the method, aiming for only two rounds. Delphi studies often start with a qualitative, open-ended first round, which was left out in this study, since the goal was primarily to prioritise materials and methods of existing diversity training courses. The teaching objectives and methods of a previously developed training programme ‘Migrants and Ethnic Minorities – Training Packages’ [32] were analysed and discussed by the authors in advance. The authors could supplement, compare and enrich the existing material with their own expertise due to their many years of research and teaching within the field of diversity competence, so that the expert panel could be provided with predefined items to be rated in the first round.

### Sample size and selection of panel of experts

To combine academic and teaching expertise with clinical, practical perspectives and experience, a panel comprised of both academic experts and health professionals was invited to participate. The inclusion criteria for academic experts were that they needed to have had – at the minimum – published on, or taught a course on, diversity (as main topic), so they could contribute to the study with in-depth research-based knowledge or with their own experience in knowledge dissemination and skills training on the topic. The health professionals (physicians and nurses) were required to be regularly encountering migrant and ethnic minority patients, to have been faced with challenges related to MEM patients, and to have experience in how to cope with those challenges.

The experts were identified through existing professional and academic European networks of the authors, internet searches and purposive snowball sampling. 89 experts (50 academics and 39 health professionals) from 20 countries were identified and invited to participate in the Delphi study. The final panel consisted of 31 experts, of whom 18 were academics and 13 health professionals. All experts (31) completed the first survey round. 26 experts completed the second round; three experts partly completed it, and two did not respond to reminders to fill out the second-round questionnaire.

### Characteristics of the panel

Of the 31 panellists, 18 were or had previously been involved in the medical care of migrant and ethnic minority patients – on average for 14 years (see Table 1 for further characteristics). 26 reported that they were involved in research activities regarding diversity. Almost half (14) had published over 10 peer-reviewed articles on the topic. Additionally, 25 were involved in the training or teaching of health professionals with regard to diversity competence/sensitivity. The panellists represented 13 European countries, including three major first-arrival countries for forced migrants/refugees (Greece, Italy and Spain). Additionally, nine panellists had migration experiences themselves; and five were born outside Europe [20].

**Table 1:** Panel characteristics

	N	%	Total (N)
**Age**			**28**
25–44 years	8	29	
45–54 years	13	46	
55–64 years	4	14	
65–74 years	3	11	
**Sex**			**31**
Male	12	39	
Female	19	61	
**Country of residency**			**26**
Austria	1	3	
Bulgaria	1	3	
Denmark	4	13	
France	2	6	
Germany	5	16	
Greece	1	3	
Italy	1	3	
Netherlands, The	3	10	
Norway	1	3	
Spain	3	10	
Sweden	5	16	
Switzerland	3	10	
United Kingdom, The	1	3	
**Educational background (multiple possible)**			**31**
BSc and BA	0		
MSc and MA	7	23	
PhD and Dr.	16	52	
MD	8	26	
**Current job position (multiple possible)**			
Administrator	2	6	
Teacher	8	26	
Nurse	2	6	
Medical doctor	8	26	
Research/academic expert	23	74	
Diversity trainer	4	13	
Other (specified in attachment)	6	19	
**Years of experience in this position**:			**29**
min. = 2 // max. = 36 // average = 14.6 years			
**(Past or present) involvement in the medical care of migrant and ethnic minority patients?**			
Yes	18	58	
No	13	42	
**Years of experience in working with migrant and ethnic minority patients**			**18**
Min. = 2 // Max. = 36 // Average = 14.4 years			
**Number of migrant and ethnic minority patients seen (by the health professionals) per month?**			**18**
0–20	7	39	
21–40	5	28	
41–60	2	11	
61–100	1	6	
More than 100	2	11	
**Involvement in research activities regarding diversity?**			**31**
Yes	26	84	
No	5	16	
**Number of publications (peer-reviewed articles, reports, books, etc.) published on diversity and/or transcultural competence topics**			**31**
None	4	13	
1–5	9	29	
6–10	4	13	
More than 10	14	45	

### First round

The first-round questionnaire was divided into three sections focusing on (A) defining diversity competence for health professionals, (B) training topics relevant for diversity competence and (C) teaching methods suited for short/online courses. Results from Section A, where panellists stated which diversity competences they deemed most important for health professionals when caring for diverse patients, are presented in another article [20]. Therefore, only Sections B and C will be presented here. The domains and items in Section B – Training Topics – were developed based on an deductive analysis of the content of all teaching materials from an existing diversity training programme to identify teaching topcis. The material was the basis for a course that lasted in total 24 h, which had been developed within a previous project on cultural competence and diversity sensitivity [32]. First, the content of all sessions were analysed and coded in a keyword-format. The study team discussed all codings and condensed them into keywords which were thematically grouped and finally arranged according to structural models of competence from a teaching perspective [33, 34] into the following domains: cognitive (knowledge), affective (reflection) or pragmatic (skills). After discussion of this analysis, in order to add missing content the authors consulted existing frameworks and guidelines developed to provide support and assistance to educators in integrating diversity competence in medical educational programmes [35–39]. Based on the final keyword list, items for a questionnaire to prioritise objectives and methods were developed and structured into domains and thematic areas according to the above-mentioned structural model of competence (see Table 2). The authors had many sessions of multidisciplinary discussions, in which they drew on their scientific socialisation, teaching experience and expertise in the fields of anthropology, politics, migration studies, health sciences and public health to refine the items, reformulate, and add explanations or examples if deemed necessary. The goal was to reach consensus on the design of all items. In the next stage a pre-test with two academic experts in diversity competence and two clinicians was conducted. After final adjustments, the first-round questionnaire was finalised: it consisted of 94 rateable items.

**Table 2:** Overview of sections, domains, and thematic areas

**Section B. Training Topic**	
**Domains**	**Thematic areas**
B.1. Knowledge	B1.1 Definitions B1.2 Specific Migrant and Ethnic Minority Population Groups B1.3 Diversity Aspects in Relation to Migrant and Ethnic Minority Population Groups B1.4 Knowledge About Migration B1.5 Policies Affecting Health of, and Healthcare for, Migrants and Ethnic Minorities B1.6 Discrimination and Inequalities Affecting Migrant and Ethnic Minority Population Groups B1.7 Public Health Issues B1.8 Medical Issues
B.2 Reflection	
B.3 Skills	B3.1 Communication Models, Techniques, and Skills B3.2 Interaction Between Health Professionals and Patients B3.3 Collaboration
**Section C. Methods**	
C.1 Teaching methods	

The main section on Training Topics (B) comprised 86 individual items divided into the three domains: Knowledge, Reflection and Skills. Under the domains Knowledge and Skills, the items were further divided into thematic areas (see Table 2). 6-point Likert scales provided the response options: very important, important, somewhat important, somewhat unimportant, unimportant, very unimportant.

The smaller section regarding Teaching Methods (C) comprised of eight items for panellists, to prioritise which methods should be recommended for the course (e.g. multiple-choice tests, case studies, video clips) using 5-point Likert scales, asking to what extent each method should be used (never, seldom, sometimes, often, predominantly).

In addition to the prioritisation of all topics, objectives and methods, the experts were asked to provide new items in an unlimited free-text field if they saw need for expansion or further important issues to be addressed in diversity training programmes. Also, a free-text field for general comments was added in case someone wanted to elaborate further on their answer.

In addition to the rateable items, the Round 1 questionnaire also contained socio-demographic questions. Written informed consent was obtained from the panellists.

### Second round

The second-round questionnaire consisted of a presentation of the first-round ratings and comments as well as a new rating round of items that were additionally suggested by the panel.

An inductive qualitative analysis was done with all suggestions and comments from the first round. For that original statements were first grouped into themes, merged if appropriate or kept in their original form, then a headline or main theme (super-category/code) was assigned to each cluster of statements, representing the content of the data In some cases statements, that were quite similar could be collapsed into one statement. In these cases the wording was still kept as true as possible to the original statements. The anonymised raw data and the final collapsed list had been shared and discussed within the study team in an iterative process, to ensure that the collapsing process did not change the meaning of the statements [40]. After this process, new items for the second-round questionnaire were developed on the basis of a final list of statements. Again, free-text fields for each thematic area were included in the questionnaire. The same rating scales as in Round 1 were used. In total, 97 new items to be rated were presented to the panel in the second round: Sections B (91) and C (6).

All results from Round 1 were presented to the panel in the form of bar-charts, grouping the six response options into three categories: 1) very unimportant/unimportant, 2) somewhat unimportant/somewhat important, 3) important/very important. First-round quotations elaborating on the rating of colleagues were also presented. This presentation came with free-text fields for every cluster of results, to allow experts to comment on the results and the former comments of colleagues. In this modified Delphi format, the panel was not asked to rate possibly disputed items from the first round again, since the main purpose was to prioritise existing teaching objectives and decide on additional items that the panel had suggested in Round 1 – to be rated in a final stage.

### Distribution of questionnaires and study schedule

The questionnaires were distributed via email, including information about the objective, the process and schedule of the study as well as a personal link to the online survey, which was set up in the latest version of SurveyXact. About three to four weeks of response time was allocated to each round. One week prior to the deadline, reminders were sent out. An additional reminder was sent one week after the deadline to those who still had not returned the questionnaire. Data were collected from 9 September 2020 to 10 January 2021.

### Analyses and reporting

Free-text statements and questions underwent an inductive, qualitative analysis. The detailed analysis process of the free-text answers is described in the methods section. The technical process was as follows: All proposed items and comments from the free-text fields were exported into Word for a manual, simplified, structuring content analysis. Individual, singular, and representative comments elaborating on ratings and results were either quoted back to the panel, to nuance quantitative data [40] or used to design new items as described above.

Standardised, quantitative items were analysed descriptively using Excel. The consensus threshold in this study was set at 80% for training topics. Therefore, to be considered a highly relevant topic, 80% of the panellists had to have rated an item as either important or very important. This high threshold was set with the goal of filtering out only the most important objectives of a vast list of topics to be included in short, possibly online, diversity training courses for health professionals [31]. For the shorter list of teaching methods, we were satisfied with a consensus level of 70%, who had to recommend a method that should be used especially in online formats.

Items rated by a 6-point Lickert scale in Section B are reported according to consensus, mean and standard deviation. Table 3 shows the top ten highest ranked topics according to mean value. Tables 4, 5 and 6 provide an overview of the items under the domains Knowledge (five thematic areas), Reflection and Skills (two thematic areas) that obtained consensus. Additionally, some of the low-rated items will be presented to show contrast to the items that reached consensus. Results from Section C are only described in the text and not presented in a table. Quotations are chosen to elaborate or nuance the panel’s ratings of the items.

**Table 3:** Highest ranked topics (according to mean value) across domains

Rank	Round	Item		Mean	SD	Consensus (%)
1	2	B3.1.7	Opportunities and best practices in working with interpreters	5.77	0.51	96
1	2	B3.1.9	Patient-centred communication with translator (how to keep the patient involved)	5.77	0.51	96
2	2	B1.1	Determinants of health	5.7	0.47	97
2	2	B1.4	Health effects of migration (pre-, during and post migration risk factors)	5.7	0.47	100
3	2	B3.1.10	Building trust in the trialogue	5.69	0.74	92
4	1	B2.13	Reflection on own stereotypes and prejudices regarding migrants and ethnic minorities	5.67	0.94	97
5	2	B3.1.16	Dangers and disadvantages of using lay interpreter	5.65	0.63	92
6	2	B3.1.11	Handling of sensible issues	5.62	0.64	92
7	2	B3.1.13	Clarification of the role of the interpreter	5.58	0.70	88
8	1	B2.3	Reflection on own habits of action, thought, emotion and evaluation	5.57	0.67	90
9	1	B1.2	Unaccompanied minors	5.55	0.66	90
9	1	B1.6	(Forms of) discrimination within the health care sector	5.55	0.94	97
10	1	B3.1.3	Specific communication techniques to find out about patient’s own explanatory model of their illness and their expectations regarding the treatment (e.g. people-centered communication, explanatory models approach)	5.5	0.99	93

**Table 4:** Training topics within the Knowledge domain ranked according to consensus

Item	Round	Question	Consensus (%)	Mean	SD
		**In your view, how important is it to include and learn about definitions and explanations of the following terms and concepts in the course?**			
B1.1	2	Determinants of health	97	5.7	0.47
B1.1	1	Refugees & asylum seekers	90	5.33	1.01
B1.1	1	Stereotypes, stigma & prejudice	90	5.48	1.07
B1.1	1	Equality, equity in relation to health & health care	90	5.48	1.01
B1.1	1	Vulnerability, vulnerability-concepts	87	5.31	1.12
B1.1	1	Discrimination	87	5.35	1.23
B1.1	1	Diversity	86	5.18	1.20
B1.1	1	Racism & xenophobia	84	5.29	1.25
B1.1	1	Migration (labour migration, forced migration, internal displacement, family reunification, etc.)	81	5.06	1.22
B1.1	1	Structural violence	81	5.23	1.13
		**In your view, how important is it to include specific information on the following population groups in the course?**			
B1.2	1	Unaccompanied minors	90	5.55	0.66
B1.2	1	Undocumented migrants	87	5.45	0.66
B1.2	1	Refugees & asylum seekers	84	5.32	1.06
		**In your view, how important is it to include and address the diversity aspects listed below in the course?**			
B1.3	1	Human trafficking	90	5.28	1.08
B1.3	1	Torture	87	5.23	1.09
B1.3	1	Living conditions (housing, working environment, etc.)	87	5.32	1.09
B1.3	1	Sexual & reproductive health, including pregnancy	83	5.07	1.08
B1.3	1	Legal status, rights & entitlements	81	5.25	1.13
B1.3	2	Social norms & values	81	5.23	0.76
B1.3	1	Socio-economic status	80	5	1.34
B1.3	1	Gender-based-violence	80	5.13	1.06
B1.3	1	Language/s & language proficiency	80	5.3	1.16
		**In your view, how important is it to include the following specific migration related aspects in the course?**			
B1.4	2	Health effects of migration (pre-, during and post migration risk factors)	100	5.7	0.47
B1.4	2	Legal rights of migrants according to migration status	89	5.26	1.06
B1.4	1	Health-related risk factors during migration process	87	5.41	0.71
		**In your view, how important is it to include the following topics in the course?**			
B1.6	1	(Forms of) discrimination within the health care sector	97	5.55	0.94
B1.5	1	National policies affecting health and health care of migrants and ethnic minorities	93	5.43	0.76
B1.7	1	Determinants of health disparities	90	5.35	1
B1.7	1	Utilization of & access to health care	90	5.42	1
B1.5	2	Local policies affecting migrants	88	5.2	0.65
B1.6	1	Structural discrimination (e.g. unequal chances in society, institutional discrimination, public and political discrimination, discrimination by law)	87	5.48	0.71
B1.5	2	National policies about health care of undocumented patients	84	5.4	0.83
B1.6	1	Medical professional code of conduct & ethics	80	5.1	1.16

**Table 5:** Training topics within Reflections domain, ranked according to consensus

Item	Round	Question	Consensus (%)	Mean	SD
		**What kinds of reflections of the health care profession should be encouraged during the course?**			
B2.13	1	Reflection on own stereotypes and prejudices regarding migrants and ethnic minorities	97	5.67	0.94
B2.8	1	Reflection on social context in which specific groups of migrants and ethnic minorities live	93	5.47	0.99
B2.11	1	Reflection on own strength in caring for migrants and ethnic minorities	93	5.4	0.99
B2.16	1	Reflection on organizational and structural factors that affect the quality of care	93	5.4	1.05
B2.3	1	Reflection on own habits of action, thought, emotion and evaluation	90	5.57	0.67
B2.12	1	Reflection on own fears and worries regarding migrant and ethnic minority patients	90	5.37	1.02
B2.5	1	Reflection on social and professional context	87	5.17	1
B2.14	1	Reflection on roles and power relations within medical encounter	87	5.37	0.8
B2.2	1	Reflection on own experiences with migrant and ethnic minority patients	86	5.31	1.12
B2.9	1	Reflection on own ability to deal with uncertainty and ambiguity	83	5.17	1.1
B2.10	1	Reflection on own ability to deal with new and unfamiliar situations	83	5.23	1.11
B2.15	1	Reflection on discrimination at their own workplace	83	5.37	0.84
B2.17	1	Reflection on possibility to implement course content at their own workplace	83	5.17	1.12
B2.1	1	Reflection on possible differences and similarities between medical encounters with migrant and ethnic minority patients and encounters with the majority population	80	5.1	1.11
B2.7	1	Reflection on (possibly different) explanatory models of disease by patients (e.g. fate, being cursed, bad karma, food induced, free radicals)	80	5.27	0.77

**Table 6:** Training topics within Skills domain ranked according to consensus

Item	Round	Question	Consensus (%)	Mean	SD
		**In your view, how important is the following topics in the course?**			
B3.1.3	1	Specific communication techniques to find out about patient’s own explanatory model of their illness and their expectations regarding the treatment (e.g. people-centered communication, explanatory models approach)	93	5.5	0.99
B3.1.5	1	Introduction to working with interpreters properly and efficiently	87	5.47	1.06
B3.1.3	1	Basic conversational techniques (e.g. respectful and sensitive communication, reflective listening, emphasizing, managing difficult situations and conflicts, negotiating)	80	5.23	1.23
B3.1.4	1	Exercises to learn to use simple lay language to explain medical content and procedures to patients	80	5.2	1.08
		**In your view, how important is the following topics in the course?**			
B3.1.7	2	Opportunities and best practices in working with interpreters	96	5.77	0.51
B3.1.9	2	Patient-centred communication with translator (how to keep the patient involved)	96	5.77	0.51
B3.1.15	2	Awareness of interpreters’ limitations (e.g. knowing medical concepts, terminology)	96	5.54	0.58
B3.1.10	2	Building trust in the trialogue	92	5.69	0.74
B3.1.11	2	Handling of sensible issues	92	5.62	0.64
B3.1.16	2	Dangers and disadvantages of using lay interpreter	92	5.65	0.63
B3.1.13	2	Clarification of the role of the interpreter	88	5.58	0.70
B3.1.12	2	Clarification of the professional role of the health care provider in an encounter with an interpreter	88	5.35	0.89
B3.1.6	2	Challenges in working with interpreters	85	5.38	0.85
B3.1.14	2	Communication with the interpreter regarding expectations towards him/her	81	5.35	0.89
		**In your view, what other actors/sectors are important for the interasectoral collaboration?**			
B3.3.2	2	Social workers	92	5.54	0.65
B3.3.15	2	Interpreters and mediators	92	5.38	0.64
B3.3.7	2	General Practioners	88	5.42	0.81
B3.3.5	2	Mental health professionals and counsellors	85	5.15	0.78
B3.3.8	2	Family and relatives	80	5.36	0.91

## Results

### Prioritisation of training topics

The 10 highest ranked topics – according to mean value across domains – are shown in Table 3. The items ‘opportunities and best practices in working with interpreters’ and ‘patient-centred communication with translator’ scored the highest. ‘Determinants of health’ and ‘health effects of migration (pre-, during- and post-migration risk factors)’ came in second. All the four highest scoring items were introduced in Round 2 by members of the panel. The one item reaching 100% consensus, since all experts deemed it to be important or very important, is ‘health effects of migration (pre-, during- and post-migration risk factors)’ which also has the lowest SD of 0.47 together with ‘Determinants of health’ indicating that these are the least disputed items.

### Prioritisation of training topics: knowledge domain

The Knowledge domain consisted of 61 items in Round 1, of which 27 reached consensus (44%) and in Round 2, six out of 34 items which were additionally proposed by the panel reached consensus (18%). Asked what definitions and concepts should be provided in a diversity training programme, the panel regarded the following items most important: ‘determinants of health’ (97%), ‘refugees and asylum seekers’ and ‘stereotypes, stigma and prejudice’ (last two 90%) (Table 4). The items ‘discrimination’ (87%) and ‘diversity’ (86%) also reached consensus whereas the item ‘culture’ (73%) did not reach consensus. The highest variability in this thematic area was found regarding the items ‘racism & xenophobia’ (SD 1.25) and ‘discrimination’ (SD 1.23). Panellists considered definitions and concepts of ‘persons of colour’ (31%) and ‘ethno-pharmacogenetics’ (26%) to be least important in planning basic diversity training programmes. One panellist made a general comment on providing definitions and concepts within a course:

*‘When teaching terminology, it is better to go beyond just terms and make the materials more interesting through cases, scenarios and examples, otherwise this part on concepts is boring for the trainees and very dry’* (AE1)^1^.

Regarding the inclusion of information about specific population groups in short courses on diversity, the highest level of importance was assigned to information about ‘unaccompanied minors’ (90% consensus), ‘undocumented migrants’ (87%), and ‘refugees and asylum seekers’ (84%). The items rated least important were ‘religious groups’ (42%) from Round 2 and ‘international students’ (25%) from Round 1. None of the new items suggested by the panel in Round 1 reached consensus (Table 4). One panellist commented, ‘*The most vulnerable population (being most discriminated [against]) should be targeted’* (AE2). Another panellist elaborated,

*‘I would only address differences when really relevant (e.g., undocumented migrants’ lack of access to the health system); in the whole training course the concept of person-centeredness should be made clear, meaning that you always have to address all specifics of that individual person, and the best way to do that is to ask the person’* (HP/AE1).

Table 4 also displays items related to the provision of knowledge on various aspects of diversity which might be relevant to consider in diverse societies, and therefore relevant to address in basic diversity courses. The panel reached consensus on the relevance of including knowledge on ‘human trafficking’ (90%), ‘torture’ (87%) as well as ‘living conditions’ (87%) within a course. Also ‘language and language proficiency’ were seen as relevant aspects to be addressed (80%) however with a large variability (SD 1.16). The lowest level of variability within this thematic area was found regarding ‘social norms & values’ (SD 0.76) which 81% of the panel found important or very important. Also ‘socio-economic status’ and ‘gender-based violence’ showed a similar level of consensus (80%), however with a wider variability range (SD 1.06–1.34). The lowest ranked diversity aspects were ‘religion’ from Round 1 which 54% ranked as important or very important training content and ‘traditional medicine’ (48%) which was proposed by a panellist and rated upon in Round 2. One panellist commented:

*‘Cultural beliefs, cultural habits and religion are all extremely important for how patients and communities understand health, what they expect when seeking healthcare, and how the eventual healthcare interaction plays out. Rather than teaching some examples of cultural habits and/or cultural or religious beliefs, I would advise taking a more open approach, […] teaching self-reflection skills and how to maintain an open and enquiring approach to patients’* (HP/AE2).

As mentioned above, the migration-related aspect ‘health effects of migration (pre-, mid- and post-migration risk factors)’ reached 100% consensus in our survey (see Table 4). Other migration-related aspects that the panel ranked highly were the items ‘legal rights of migrants according to migration status’ (89%) and ‘health-related risk factors during migration process’ (87%). The lowest scores were given to two items in Round 2: ‘circular migration’ (41%) and ‘historical perspectives of migration’ (38%). One of the panellists nuanced his/her own response in this thematic area:

‘*I would rank push and pull factors, current migration flows and circular migration as not important for those involved in the direct care of migrants. However, these are important topics for lobbying in policy change and for epidemiology’* (HP1).

Within the thematic area ‘Policies, Discrimination and Public Health issues’, Table 4 shows that the item ‘discrimination within the healthcare sector’ was highest ranked, with 97% of the panel agreeing that it is important or very important to include in a course. A panellist wrote that, *‘[it] […] is a neglected problem (and a taboo among many care providers who like to think that they deliver equal care to every patient) and therefore needs a lot of attention’* (AE3).

Also *‘*national policies affecting health and healthcare of migrants’ (93%) and ‘determinants of health disparities’ (90%) were ranked highly. One panellist advised keeping the policies content ‘*…short and practical, otherwise the topic can get very general and theoretical and lose the attention of the doctors’* (AE1).

However, the *‘*medical professional code of conduct’ also reached consensus with 80% of the panel agreeing that it is important or very important. Among the lowest ranked items in this thematic area were, ‘conventions against torture’ and ‘Convention on the Rights of the Child’ but still 54% of the panellists agreed that these items are important or very important to include in the course.

### Prioritisation of training topics: reflections domain

There was much unity regarding the importance of participants being invited to undertake Reflection during diversity training courses. Out of 18 questions on reflection provided in Round 1, only three (17%) did not reach consensus, along with four related topics additionally suggested by the panel in the second round. The item that reached the highest score was ‘reflection on own stereotypes and prejudices regarding migrants and ethnic minorities’ (consensus 97%, mean 5.97, SD 0.949) (see Table 5). The second highest rated items were ‘Reflection on own strength in caring for migrants and ethnic minorities’ (93%) and ‘Reflection on organisational and structural factors that affect the quality of care’ (93%). Additionally, ‘Reflection on possibly different explanatory models of disease by patients (e.g. fate, being cursed, bad karma, food-induced, free radicals)’ also reached consensus (80%). The item ‘Reflection on biomedical explanatory models (theoretical medicine) of health and illness’ received the lowest score in Round 1 (63%), and the item ‘Reflection of own experiences as a member of an ethnic minority group’ suggested by the panel and rated in Round 2 only reached 58%. One panellist stressed the importance of including reflection in a course on diversity competence: *‘This part of the training is the most important in my view and should get the most attention/time’* (HP/AE1).

### Prioritisation of training topics: skills domain

Out of five questions in the thematic area ‘Communication Models, Techniques and Skills’ in Round 1, four reached consensus. The highest rated item was ‘specific communication techniques to find out about the patient’s own explanatory model of their illness and their expectations regarding the treatment (e.g. people-centred communication, explanatory models approach)’ which 93% of the panel scored as very important or important. Also, the items ‘Introduction to working with interpreters properly and efficiently’ (87%) and ‘basic conversational techniques (e.g. respectful and sensitive communication, reflective listening, emphasising, managing difficult situations and conflicts, negotiating)’ (80%) reached consensus (See Table 5). The only item that did not reach consensus was ‘basic communication models (encoding, decoding, feedback, co-creation of meaning, four-side model and the like)´ (69%), although it was still rated highly by the panel. One panelist commented, *‘I think good communication is more a question of attitude, and of continuous work with that, than a question of techniques’* (HP/AE3).

When asked to rate on the most important skills in working with an interpreter 96% of the panel scored three items as important or very important: ‘Opportunities and best practices in working with interpreters’, ‘patient-centered communication with translator (how to keep the patient involved)’ and ‘awareness of interpreters’ limitations (e.g. knowing medical concepts, terminology)’ – all showing a consensus of 96% and low variation (see Table 6). Out of the 11 items about interpreters in Round 2, only one did not reach consensus: ‘organisation of the cooperation of interpreter services’ (73%).

Table 6 also shows which actors/sectors the panel considered important for intersectoral collaboration, and which therefore should be thematised in short courses. Out of the 18 suggested collaborators, only five reached consensus. Social workers, interpreters and mediators all reached the same highest score: 92%. General practitioners (88%) were also considered important collaborators. The lowest ranked collaborators were ‘police’ (28%) and ‘cultural associations’ (28%).

### Prioritisation of teaching methods

Eight teaching methods were presented to the panel in Round 1, to be prioritised. Four of them were highly recommended by our panel: videos with examples of interactions (97%), case studies (97%), portfolio questions (70%), and short lectures on specific topics (maximum 10 min) (70%). In the second round four out of six further methods, additionally suggested by the panel, were highly recommended to be used in diversity training courses: ‘migration narratives (case studies)’ (92%), ‘practical exercises’ (88%), ‘role plays (of cases)’ (85%) and ‘examples of illness narratives’ (80%). The methods that were least recommended were ‘multiple-choice exercises’ (27%) and ‘multiple-choice tests (to test if content was understood and remembered correctly)’ (30%). One panellist stressed that *‘more interactive methods are always wanted […]’* (AE1). Another panellist explained:

‘*The teaching methods must reflect the subject. Since there are very few simple straight answers on “rights” or “wrongs” in this field, the teaching methods must open up [space] for reflection, development and complications’* (HP/AE3).

## Discussion

Our findings point to key priorities in the education of health professionals related to diversity competence within the format of a short/online course. These should be information on determinants of health, combined with reflections on the social contexts in which patient’s live, national policies that might affect their care, as well as organisational and structural factors that might affect the quality of care. Participants should learn about possible health effects of migration and related risk factors. Additionally, they should be provided with the opportunity to think about discrimination within the healthcare sector, and reflect on their own stereotypes and prejudices as well as their own strengths in caring for migrants and ethnic minorities. Diversity training courses should also help to develop skills to work with interpreters in a professional manner and find out about the explanatory models and expectations of patients. As far as didactic approaches are concerned, our findings suggest that diversity competence training should work with realistic example material and should be based on reflective exercises and activities.

Our results showed a trend towards more focus on ‘diversity’ and less focus on ‘culture’. For example, the question of whether a definition of ‘culture’ should be provided in trainings did not reach consensus, whereas our panel considered ‘diversity’ to be an important topic. This is interesting in the light of a recurring focus on culture, multiculturalism, cultural differences, and cultural assimilation in the public debate related to migrants [41]. However, this result is in line with literature from recent years criticising the cultural competence concept for both its strong focus on culture and the misrepresentation of the concept [42–44]. It might suggest a theoretical shift away from essentialising concepts, towards multicollectivity [15], the diversity within diversity [1] and intersectionality [19] with the implications to take other aspects of a patient’s identity into account, promoting individualised, patient-centred provision of healthcare [16].

The high level of consensus for items referring to reflection on own stereotypes, prejudices, habits, and organisational and structural factors that can affect the quality of care, corresponds well with Kumagai and Lypson [45] who argue that medical education should focus on developing and fostering a critical consciousness in medical students, instead of a more traditional knowledge-based competence development.

In general, there was a high level of consensus regarding the reflection items. Affective competence development is a challenging task to be fostered, especially in short and/or online courses. In short courses, there might be a temptation to pack as much knowledge content as possible into the training, to provide participants with information on what trainers consider relevant and basic, reducing time for contemplation, individual or intersubjective discussion and analysis of experiences and affects. The online format is also commonly perceived to be mainly based on one-way communication with very few options for dialogue and interaction [46]. A study exploring the effect of online learning on students’ engagement found that online students were less likely to engage in collaborative learning and discussions; and the students also reported lower quality of interactions [47]. However, particularly regarding reflective activities, some studies suggest the opposite: that online learning formats work well [48] and that they can even be especially useful for expressing difficult emotions related to racism, prejudice, and discrimination [49]. Consequently, a well-planned curriculum that provides time and space for an individual and collaborative confrontation and analysis of affects and attitudes, is necessary to adequately prepare for the development of affective competences. Basic training courses should be moderated by prepared, skilled, experienced, empathic, and non-judgemental trainers to achieve the learning objectives.

Another thematic area which received much attention with high consensus and top scores was that of ‘Communication Models, Techniques, and Skills’ and in particular working with interpreters and dealing with the challenges that health professionals perceived as related to this collaboration. A Delphi study focusing on health services and the treatment of immigrants [50] conducted in 2012 concluded that interpretation was one of the most important issues in providing healthcare to migrants and ethnic minorities. Another study from 2017 exploring if diversity competences are implemented in European Medical education programmes, showed that the topic “working with an interpreter” was not included in most curricula [25]. This suggests that European Medical education programmes are not developing quickly enough and therefore might not be front-runners when it comes to adjusting to societal needs. This also means that, to date, a knowledge gap still exists when it comes to health professionals’ knowledge of working with interpreters. To close this gap, it would be relevant and appropriate to include the topics ‘Introduction to working with interpreters properly and efficiently’, ‘patient-centred communication with translator (how to keep the patient involved)’ and ‘awareness of interpreters’ limitations’ in curricula in both short and online courses for health professionals.

Some of the more traditional methods that reached consensus in this study (short lectures, portfolio questions) are a good match for the more knowledge-based topics. However, many of the topics which reached consensus focused on the student’s ability to understand how a person’s social position and experiences of advantage or disadvantage shape the person’s understanding of the world. This seems to require skilled teachers who are open to innovative approaches and novel methods adapted to short courses and online learning. Some of the methods that our panel recommended, such as ‘role plays (of cases)’ and ‘practical exercises’ are challenging tasks. Teachers have to be able to design practical exercises for students of different educational backgrounds, working in various different contexts, knowing the clinical reality will be complex and unique in every situation so there might be no fitting textbook examples of interactions. Furthermore, using such interactive methods demands advanced moderation skills to ensure that the outcomes work in the direction of desired teaching objectives.

Population diversity is a relevant issue to be addressed in the basic and continued education of health professionals. There are wide-ranging lists of skills to be fostered and therefore extensive training programmes to be provided. Curricula of professional education are already substantial and there are time constraints within the busy schedules of health professionals, whose priorities might also lie with other topics. The provision of short or online courses opens up the possibility of encouraging health professionals to enhance both their professional and personal development by learning how to handle diversity – thereby attracting participants who would otherwise not have considered undertaking such courses. While short courses will, inevitably, not address all potentially relevant objectives or provide guidance to deal with all the daily challenges that health professionals face in caring for migrant and minority patients, these courses can provide a valid entry point in fostering the ability of health professionals to take good care of all patients in today’s plural societies. This study sets out to provide support for stakeholders and trainers in the design of short and online diversity competence courses so as to broaden the reach of these courses within the large group of health professionals who need basic training to provide inclusive healthcare in increasingly diverse populations.

### Strengths and limitations

Due to the selective sampling of panellists, we do not claim our expert panels’ prioritisations to be representative: the results from the Delphi study could be specific to this panel. However, their prioritisations generally align well with recent literature on competence requirements among healthcare professionals, and with latest international guidelines on the training of healthcare professionals regarding migration and ethnic minority health. Furthermore, the relevance of our findings is also supported by the inclusion of experts from a variety of countries with quite different migration policies, migration flows, and roles as countries of first arrival, transit and/or destination.

The panel consisted of both academic experts and health professionals who could provide reliable professional assessment of training topics and teaching methods for short/online courses. The academic experts added value through their research and teaching experience in diversity competence. Through their participation in the study the academic experts were invited to reflect upon the importance of learning objectives and content well known to them, and thereby contribute to a collective development of the field of diversity competence training. The health professionals’ experience with migrant and ethnic minority patients enabled them to assess the relevancy of certain skills and knowledge in their everyday professional lives, ensuring that essential competences for clinical practice will be fostered in short training courses. Due to the particular focus of this study being related to migrant and ethnic minorities, the experts – both academic and health professionals – were chosen specifically due to their expertise and/or experience in this field. Had the study emphasised the whole diversity spectrum and for example included experts from the field of LGBTQIA + health, the results might have fallen out differently due to difference in priorities and needs.

A further limitation might be that all panellists were living in Europe and presumably had a European perspective on diversity competence. Therefore, a relevant question to put forward could be whether our results would be applicable in a global setting. Some of the panellists, being migrants themselves, add their experiences from other parts of the world to our findings. We could argue this adds a global perspective to our results. Comparing the results from this study to global guidelines on diversity competence standards, such as those provided by the WHO [24], our results show the same trends when it comes to an increased focus on diversity, social determinants of health, awareness of stereotypes and bias, and also in using reflective exercises and activities as teaching methods. Our results seem therefore to be applicable in a more global setting.

This study used an adapted Delphi method consisting of two rounds with predominantly predefined items. Delphi studies most commonly consists of three rounds with a fully open first round without predefined items and multiple rating rounds of the same items. However, due to the already quite substantive literature and recorded experiences on the subject, as well as our own training expertise we decided to leave out an open, initial round, since we could already present a series of suggestions to be rated upon in a standardised format, making two rounds seem sufficient.

## Conclusion

This study provides a prioritisation of diversity training content for curriculum development for short and/or online courses for health professionals and can thereby help reduce course content, fostering professional development and enabling diversity competence training courses to be implemented also in cases where time or financial resources are limited. With the benefit of their wide and varied professional experience, the panellists proposed and then rated topics within the domains of: knowledge, reflection and skills of special relevance for diversity trainings, and suggested teaching methods that could be particular useful for enhancing the diversity learning objectives. The panellists also pointed to the need for giving high priority to the didactic and technical design of short or online courses to support and enable reflective learning. This may require additional competences of the teachers and more effort on the side of course providers and teachers to initiate, moderate and monitor, targeted learning processes, but with clear potentials for providing important long-term benefits for the learners. Further research with larger samples may be needed to validate the results. Subsequent steps could include further validation, development and evaluation of curriculum content and teaching methods in e-learning frameworks by setting up experimental courses in different settings with well-planned evaluation designs documenting outcomes for participants and patients, and – in the long-term – healthcare provision offering greater inclusivity for migrant and ethnic minorities.

## Data Availability

The datasets used and/or analysed during the current study are available from the corresponding author on reasonable request.

## Abbreviations

MEM: Migrants and ethnic minorities
CME: Continuous medical education

## Footnotes

1 Acronyms only refer to professions, without individual assignment: HP = Health Professional, AE = Academic Expert, HP/AE in cases where the panellist is both
